# Calcium-Free Dialysate Hemodialysis: A Simplified Approach

**DOI:** 10.3390/jpm14060660

**Published:** 2024-06-20

**Authors:** Alexandra Corbu, Florian Terrec, Paolo Malvezzi, Arnaud Jouzier, Thomas Jouve, Lionel Rostaing, Hamza Naciri Bennani

**Affiliations:** 1 Nephrology, Haemodialysis, Apheresis and Kidney Transplantation Department, Grenoble University Hospital, 38043 Grenoble, France; acorbu@chu-grenoble.fr (A.C.); fterrec@chu-grenoble.fr (F.T.); pmalvezzi@chu-grenoble.fr (P.M.); ajouzier@chu-grenoble.fr (A.J.); tjouve@chu-grenoble.fr (T.J.); hnaciribennani@chu-grenoble.fr (H.N.B.); 2 Univ. Grenoble Alpes Inserm U 1209, CNRS UMR 5309, Team Epigenetics, Immunity, Metabolism, Cell Signaling and Cancer, Institute for Advanced Biosciences, 38000 Grenoble, France

**Keywords:** hemodialysis, dialysate, calcium-free, circuit anticoagulation, citrate, calcium reinjection, heparin-free

## Abstract

Intermittent hemodialysis (HD) in high-bleeding-risk patients presents a challenge as circuit anticoagulation using heparin is contraindicated in such cases. Recently, the use of calcium-free citrate-containing dialysate with calcium supplementation emerged as a viable alternative to heparin-circuit anticoagulation. This is a retrospective, monocentric study to evaluate dialysis efficacy using calcium-free citrate-containing dialysate with calcium reinjection into the venous line in hemodialysis patients at risk of bleeding. A total of 53 patients were analyzed: 52 had a temporary contraindication to systemic anticoagulation (active bleeding or surgical intervention), and 1 chronic HD patient had prolonged bleeding time due to inoperable arteriovenous fistula stenosis. Only 7 out of 79 dialysis sessions performed were prematurely terminated (vascular access dysfunction). The median dialysis time was 240 min (range: 150–300). The chronic dialysis patient had 108 sessions with no premature termination. Frequent monitoring of ionized calcium was performed throughout the dialysis sessions: levels remained stable at T0 and T + 60 min (1.08 ± 0.08 mmol/L) and slightly increased at the end of the dialysis session (1.19 ± 0.13 mmol/L), remaining within normal limits. Target postfilter ionized calcium <0.4 mmol/L was achieved in all sessions (0.31 ± 0.07 mmol/L). There were no cases of symptomatic hypo-/hypercalcemia and no need for calcium infusion rate adjustment throughout the sessions. Hemodialysis with calcium-free citrate-containing dialysate and calcium reinjection into the venous line is efficient and safe in HD patients with contraindications to systemic anticoagulation.

## 1. Introduction

Intermittent hemodialysis necessitates appropriate anticoagulation to prevent extracorporeal circuit coagulation without blood loss and to achieve adequate blood clearance of uremic toxins.

Unfractionated heparin or low-molecular-weight heparin remain the primary anticoagulants utilized in hemodialysis. However, their administration typically results in systemic anticoagulation, thereby impeding their use in patients at high risk of bleeding. Over the years, alternatives to heparin anticoagulation have been developed to ensure adequate dialysis in patients with temporary or permanent contraindications to anticoagulants (such as active bleeding, recent surgery, or pericardial effusion).

Iterative saline flushes during hemodialysis can be employed in such cases, but their efficacy is questionable, with this technique being associated with a high risk of clotting and volume overload. Previous studies have shown varying results regarding their efficacy, with circuit coagulation rates of up to 50% [1,2,3,4,5].

Specific heparin-coated membranes may also serve as an alternative to saline flushes, as they have been found to have similar efficacy [6,7,8]. However, they are prone to premature coagulation of the circuit, often necessitating a new circuit setup during the dialysis session to complete the treatment.

Regional heparin anticoagulation with heparin injected into the arterial line and protamine into the venous line is a cumbersome technique for a chronic dialysis center and has been shown to increase the risk of bleeding after dialysis, making it an inadequate alternative in high-bleeding-risk patients [9,10]. The use of a citrate dialysis bath replacing acetate has also been explored, but it did not allow for a complete dialysis session without anticoagulation [11,12,13].

Regional citrate anticoagulation, achieved by infusing citrate into the arterial line and calcium into the venous line, has been widely used in intensive care units to achieve isolated anticoagulation of the extracorporeal circuit without increasing the bleeding risk. However, as it requires infusions of citrate and calcium, it poses technical challenges for the dialysis team. Other disadvantages include contraindications in patients with liver disease and potential adverse effects such as sodium retention, volume overload, metabolic alkalosis, hypotension, or arrhythmias [14,15,16,17,18,19].

Therefore, it is crucial to explore new anticoagulation strategies to maintain blood circulation throughout the extracorporeal circuit with minimal bleeding risk for high-risk patients.

A technique of heparin-free anticoagulation using a calcium-free, citrate-containing dialysate with calcium reinjection into the venous line has shown promising results in recent years [20,21,22,23], allowing for proper anticoagulation of the circuit with minimal impact on patients’ ionized calcium levels (iCa). The absence of calcium in the dialysate creates a significant diffusive gradient, facilitating the transfer of calcium from the blood to the extracorporeal system, thereby preventing coagulation.

The aim of our study was to evaluate the efficiency and safety of this technique, employing a protocol of calcium-free citrate-containing dialysate with calcium reinjection into the venous line in an acute hemodialysis center.

## 2. Materials and Methods

We conducted a retrospective, observational, monocentric study in our Apheresis and Hemodialysis Center between January and October 2023. We included 53 patients who required heparin-free hemodialysis. Patients requiring platelet antiaggregant therapy were excluded.

### 2.1. Dialysis Parameters

All hemodialysis sessions were performed using a B-Braun Dialog+^®^ dialysis machine (B.Braun Avitum AG, 34209, Melsungen, Germany) and high-flux dialyzer Nipro ELISIO™ (Nipro Medical Europe, 2800, Mechelen, Belgium).

We utilized a calcium-free citrate-containing dialysate (CITRASATE^®^ sans calcium B484 HEMOTECH, Toulouse, France) with the following final composition after mixing with sodium bicarbonate concentrate: potassium 2 mmol/L, glucose 1 g/L, citric acid 0.8 mmol/L, acetic acid 0.3 mmol/L, calcium 0 mmol/L, magnesium 0.5 mmol/L, sodium 140 mmol/L, and bicarbonate 31–35 mmol/L. The dialysate flow rate was fixed at 500 mL/min, and the dialysate temperature was set at 36.5 °C.

We maintained a blood flow rate of 250–350 mL/min, and ultrafiltration was adjusted based on the patient’s volume status and dry weight. Isolated ultrafiltration was contraindicated.

### 2.2. Calcium Rejection

For calcium restoration, we employed a 10% calcium chloride solution with a concentration of 0.1 g/mL, administered at a standard continuous perfusion rate of 42 mL/h. This rate was demonstrated to induce a plasma calcium concentration equivalent to that obtained with a dialysate containing a calcium concentration of 1.5 mmol/L for a dialysis clearance of 210 mL/min (Hemodialysis Protocol with a Calcium-Free Dialysate, Grenoble University Hospital, see Supplementary Data). The calcium solution was administered through a continuous intravenous electric syringe, with reinjection directly into the venous return line after the venous bubble trap, through a Y-shaped connector between the venous needle and the venous line.

The target patient ionized calcium (iCa) (prefilter) was set at 1.10–1.25 mmol/L, and the machine iCa (postfilter) was set at 0.15–0.4 mmol/L. The objective was to maintain the total serum calcium concentration within the normal range (2.2 to 2.6 mmol/L). Blood for patient iCa was drawn from the arterial line before the blood pump, and for machine iCa, from the venous line postfilter before reperfusion with ClCa2. Repeated calcium measurements were obtained during the dialysis session, including patient iCa and total calcium (tCa) at the beginning (T0) and end of the session, and patient iCa and machine iCa after the first hour (T1).

### 2.3. Session Parameters

Clinical parameters such as blood pressure and heart rate were recorded hourly throughout the session. Patients were closely monitored for signs or symptoms of hypo- or hypercalcemia, with emergency ionized calcium measurements performed in such cases. Dialysis parameters including arterial, venous, and transmembrane pressures, as well as Kt/V, were recorded by the dialysis machine. Blood clot formation was assessed by nurses using a visual scale after flushing the dialysis circuit at the session’s end with normal saline (Global Thrombosis Index, see Supplementary Data—Table S2) [20].

### 2.4. Data Collection and Statistical Analyses

Hemodialysis, clinical, and biological data were collected from electronic medical records (CristalNet software V01.04.02.43 and Easily software V12.00.12). Statistical analyses were conducted using Excel 2016 software. Quantitative variables are presented as means ± standard deviations (SD) or medians with quartiles (Q1–Q3), while qualitative data are presented as numbers and percentages. The chi-squared test was used for categorical variables, and the Wilcoxon or Kruskal–Wallis test were used for continuous variables. A two-sided *p*-value of <0.05 was considered statistically significant. Statistical analyses were performed using R statistical software (version 4.4.0).

The study was conducted in accordance with the Declaration of Helsinki guidelines and approved by CNIL (French National Committee for Data Protection; approval number 1987785v0). The biobank collection number is BRIF BB-0033-00069. Informed consent was obtained from all subjects participating in the study.

## 3. Results

Our analysis comprised 52 patients with a temporary contraindication to systemic anticoagulation who required hemodialysis in our acute hemodialysis center and 1 chronic male patient, 58 years old, from our center with prolonged compression time of the arteriovenous fistula (sometimes exceeding 45 min), even when very low doses of heparin were used, due to a tight stenosis that could not be surgically corrected. The main comorbidities of the patients were arterial hypertension (82%), type II diabetes (43%), peripheral artery disease (28%), and ischemic heart disease (22%).

Among the 52 patients, a total of 79 sessions were conducted. The sex ratio M/F was 33/19 and the mean age was 65 ± 12 years. The patient and dialysis parameters are summarized in Table 1. The primary indication for heparin-free dialysis was recent surgery (49% of cases), followed by active bleeding (47% of cases) and elective organ biopsy (4% of cases). The median duration of a dialysis session was 240 min (range: 150; 300), and the median blood flow was 300 mL/min (range: 200; 380). The mean Kt/V was 1.42 ± 0.23. Clotting events occurred in 7 out of 79 sessions (9%), primarily due to vascular access dysfunction or rarely filter coagulation. No incidents were recorded during the dialysis sessions (such as symptoms of hypo- or hypercalcemia, malaise, or cramps).

**Figure 1:**
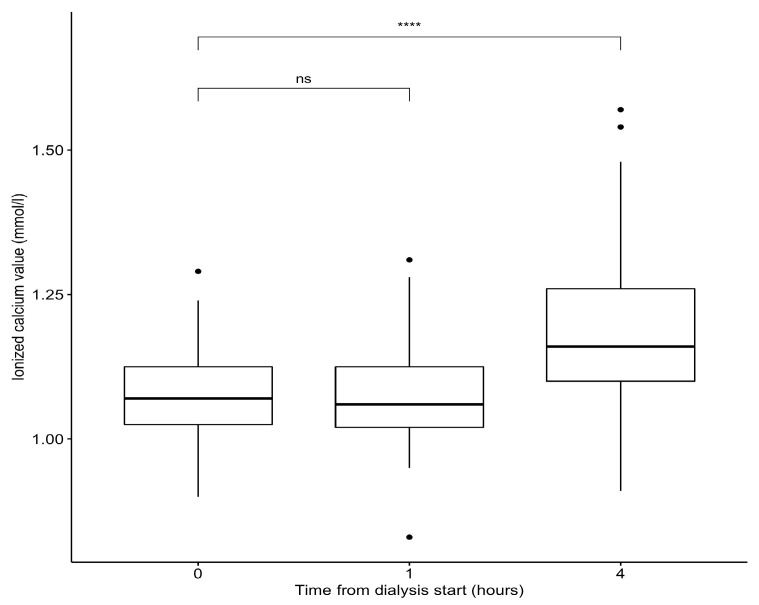
Variation in prefilter ionized calcium values during dialysis sessions. Abbreviations: ns= nonsignificant. Significance levels: **** for *p* < 0.0001.

**Figure 2:**
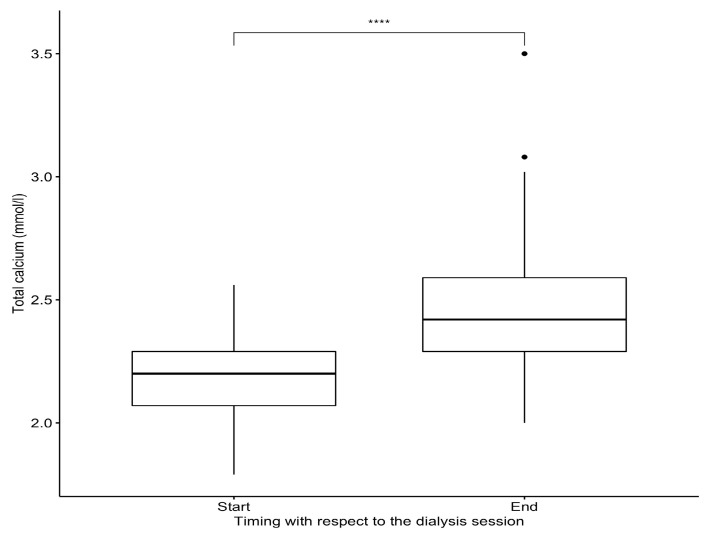
Variation in total calcium at T0 and end of dialysis session. Significance levels: **** for *p* < 0.0001.

Recorded ionized calcium (iCa) levels throughout the sessions are shown in Figure 1. The mean prefilter iCa at T0 was 1.08 ± 0.08 mmol/L, remaining stable at T1, and reaching 1.19 ± 0.13 mmol/L at the end of the dialysis session (Table 2). The mean total calcium (tCa) was 2.18 ± 0.16 mmol/L at T0 and increased to 2.46 ± 0.26 mmol/L (*p* < 0.05) by the end of the session (Figure 2). The mean postfilter iCa was 0.31 ± 0.07 mmol/L.

**Figure 3:**
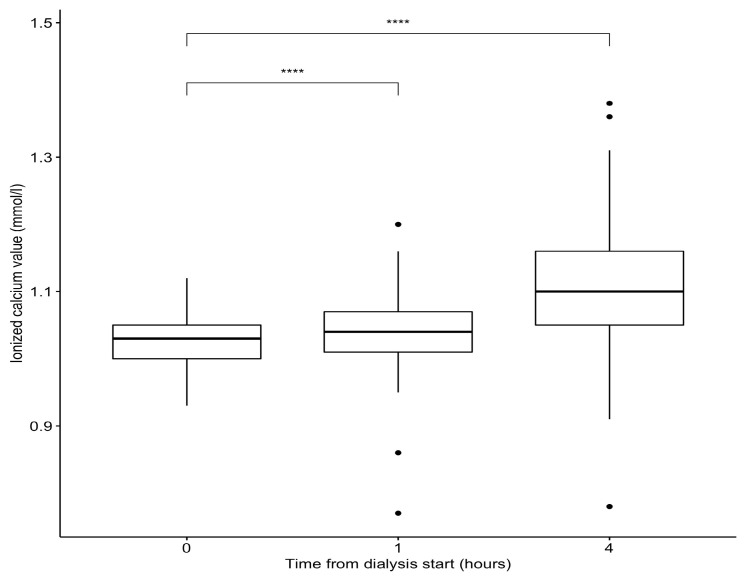
Variation in ionized serum calcium for our chronic hemodialysis patient. Significance levels: **** for *p* < 0.0001.

**Figure 4:**
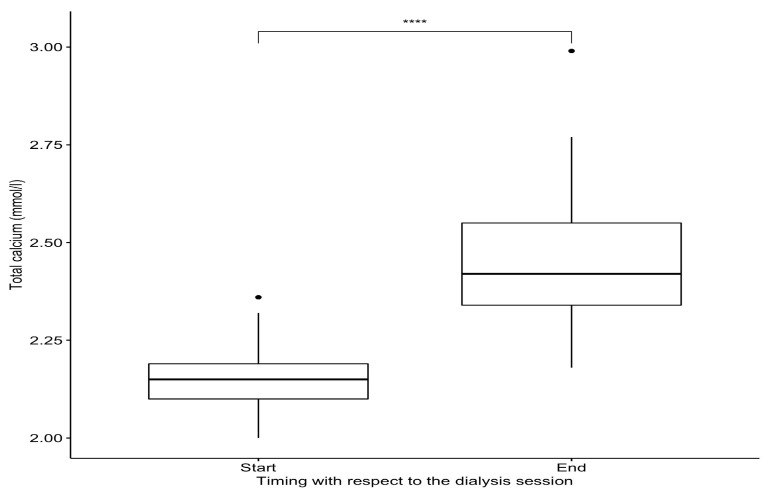
Variation in total serum calcium for our chronic hemodialysis patient. Significance levels: **** for *p* < 0.0001.

Regarding our chronic patient, the use of a calcium-free dialysate resulted in a shorter compression time (less than 10 min). Over the study period, he underwent 108 complete 4 h hemodialysis sessions with an average Kt/V of 1.36 ± 0.12. The dialysate flow rate was 500 mL/min, and the median blood flow rate was 300 (range: 250; 350). Total ultrafiltration averaged 2.3 ± 1.2 L per session. No incidents were recorded during the dialysis sessions. The variations in ionized and total calcium between the start and end of the hemodialysis sessions are presented in Table 3 and Figure 3 and Figure 4.

## 4. Discussion

In our investigation, we evaluated the efficacy and safety of the calcium-free dialysis technique with a constant rate of calcium reinjection into the venous line at 42 mL/h using a calcium chloride solution with a concentration of 0.1 g/mL. Of the 79 sessions conducted in 52 patients, only 9% were prematurely terminated primarily due to vascular access dysfunction. Regarding our chronic dialysis patients, 108 sessions were completed without premature termination. These results align with prior studies, emphasizing the imperative of robust vascular access when employing this dialysis technique.

The technique’s safety was affirmed through meticulous calcium level monitoring. Baseline ionized and total calcium levels were utilized at session onset to calibrate maintenance calcium infusion. Monitoring of pre- and postfilter iCa (patient and machine) at specific intervals was undertaken to assess calcium diffusion dynamics, particularly during the critical initial hour of dialysis.

The prefilter ionized calcium remained largely unchanged between T0 and T + 60 min (1.08 ± 0.08 mmol/L), with a slight increase observed by session conclusion (1.19 ± 0.13 mmol/L), remaining within the normal range. Incidences of asymptomatic hypo- or hypercalcemia were infrequent. The calcium infusion rate remained constant at 42 mL/h throughout, requiring no adjustment.

Patients presenting with a high risk of bleeding are frequently encountered in acute hemodialysis centers, posing a formidable challenge to the dialysis team when conventional anticoagulation is contraindicated. Recent years have witnessed a surge in interest towards identifying alternatives to heparin anticoagulation for optimizing dialysis efficiency. Diverse alternatives to dialysis circuit anticoagulation have been explored, encompassing methods such as circuit rinsing with isotonic saline, pre-dilution hemodiafiltration, and the adoption of heparin-coated membranes. The efficacy of these methods varies across studies, with reported efficiencies ranging between 50% and 75% [1,2,3,4,5,6,7,8].

Regional heparin anticoagulation, entailing the administration of heparin prefilter and protamine postfilter, has been employed, albeit with a notable risk of rebound anticoagulation. The challenge lies in the intricate adjustment of protamine dosage, especially given its shorter half-life compared to heparin [9,10].

Subsequent innovations introduced alternatives employing citrate either in the dialysis bath or directly into the extracorporeal circuit. Utilization of a citrate dialysis bath, in lieu of acetic acid, tends to reduce ionized serum calcium levels, albeit remaining above the threshold of 0.4 mmol/L required for comprehensive anticoagulation. This method mitigates pore clogging, consequently reducing heparin requirements by approximately 50%. However, heparin supplementation is often indispensable to ensure the completion of dialysis sessions [11,12,13].

Furthermore, regional citrate anticoagulation, achieved by citrate perfusion into the arterial line with calcium supplementation into the venous line, has emerged as the gold standard treatment in cases where heparin is contraindicated. This approach has exhibited favorable outcomes in terms of efficacy and tolerance, particularly in continuous renal replacement therapy (CRRT) settings [14,15,16]. However, its adaptation to intermittent hemodialysis for chronic hemodialysis patients has yielded variable efficacy, beset by laborious implementation due to fluctuating citrate and calcium levels and the risk of systemic citrate accumulation leading to hypocalcemia or metabolic alkalosis [16].

Consequently, a novel simplified technique involving a calcium-free citrate-containing dialysate with calcium reinjection into the venous line based on ionic dialysance has emerged, yielding promising results [20,21,22,23]. The absence of calcium in the dialysate forestalls coagulation within the dialyzer and the circuit when ionized serum calcium falls below 0.4 mmol/L [16]. Calcium clearance mirrors ionic dialysance, akin to urea clearance, facilitating precise calculation of the requisite calcium for administration in the return line. This technique precludes the use of isolated ultrafiltration due to the absence of a diffusion process and consequent lack of calcium clearance. Should isolated ultrafiltration be necessary, it can be administered at the session outset, just preceding calcium infusion.

In a study by Faguer et al. [20], encompassing 35 intensive care unit patients, 101 sessions of intermittent hemodialysis without anticoagulation employing a calcium-free citrate dialysis bath were conducted. The median dialysis session duration was 294 min [240; 300], with a median ultrafiltration of 2.3 L [1; 2.8] and an average Kt/V of 1.11 ± 0.4. Calcium solution reinjection, initiated at an initial flow rate of 60 mL/h for 30 min, was subsequently adjusted according to ionic dialysance. The average calcium reinjection amounted to 78.4 ± 18.7 mmol, with an average ionic dialysance of 185 ± 42 mL/min. Of the 101 sessions, only three cases of circuit coagulation were recorded (at 30, 60, and 210 min), attributed to catheter malfunction, with no post-dialysis hemorrhagic events within 24 h. Postfilter ionized calcium dosage remained stable 60 min and 180 min after dialysis initiation, with values of 0.35 ± 0.17 mmol/L and 0.38 ± 0.14 mmol/L, respectively (*p* > 0.05). Prefilter ionized calcium levels persisted within physiological limits throughout the session, and the total calcium/ionized calcium ratio remained constant from session onset to conclusion.

The absence of serum citrate accumulation with the employment of a calcium-free citrate dialysate was corroborated by a single-center retrospective study by Medrano et al. [23]. Among 908 conducted sessions, only 20 (2%) were unsuccessful due to clotting events. The duration of dialysis sessions exceeded 4.5 h in 135 cases (15%), with a maximum duration of 6 h. The citrate concentration in the calcium-free citrate-containing dialysate stood at 0.8 mmol/L. Although serum citrate concentration rose during the dialysis session, it remained within the normal range in nearly all instances, with normal serum concentrations restored one hour post-dialysis. Similarly, the total/ionized calcium ratio exhibited minimal variation, remaining below 2.2 in all patients. The median postfilter iCa concentration was 0.31 mmol/L [0.13; 0.89]. These findings underscored that the reduction in calcium levels below 0.4 mmol/L stemmed not solely from the low citrate dose in the dialysate but rather from calcium elimination through the filter.

The same authors explored a dialysate devoid of calcium or citrate, featuring an acetate concentration of 2 mmol/L in 19 patients. No premature session terminations were reported, with postfilter ionized calcium concentrations remaining below 0.5 mmol/L one hour post-dialysis.

Scarfogliere et al. [21] further validated the efficacy and safety of this hemodialysis technique in a retrospective study involving 21 patients, conducting a cross-over comparison of dialysis sessions employing calcium-free citrate dialysate versus heparin. A dialysis session success rate of 94% was noted with calcium-free citrate dialysate compared to 97% with heparin (*p* = 0.22). Circuit loss rates were 1.5% versus 0.5% (*p* = 0.23), with a statistically significant difference observed in the average session duration between the two groups (242 ± 17 min versus 237 ± 8 min, *p* = 0.004). No instances of hypocalcemia or cardiovascular complications were reported during dialysis sessions employing calcium-free citrate dialysate. Mean pre-dialysis serum calcium levels did not significantly differ between groups (2.35 ± 0.12 versus 2.34 ± 0.1 mmol/L, *p*= 0.37).

Finally, a single-center prospective study by Robert et al. [22] investigated 29 dialysis sessions with a calcium-free citrate dialysate among 17 patients. It was demonstrated that calcium perfusion rate adjustments during the session based on repeated prefilter ionized calcium dosages at various time points were unnecessary. Only 2 sessions out of 27 were terminated prematurely, attributable to vascular access issues.

A noteworthy observation in our study was the significant increase in prefilter total serum calcium between session commencement and conclusion for our cohort of 53 patients. This asymptomatic rise, often within normal thresholds, likely emanated from progressive clotting of dialyzer fibers at the blood inlet, impeding blood flow and diminishing exchange surface area. Consequently, fiber coagulation engendered a gradual reduction in ionic dialysance and a marginal serum calcium uptick with sustained calcium perfusion rates. Fiber coagulation manifestations, discernible via fiber whitening indicative of retrofiltration, were observed.

As previously demonstrated, achieving a postfilter iCa at T1 below 0.4 mmol/L serves as a yardstick for optimal diffusion dynamics to prevent clotting within the dialysis membrane. All sessions in our study met this criterion, underscoring the technique’s efficacy.

This technique holds promise for chronic hemodialysis patients, as evidenced by our findings. In a case involving a patient with an arteriovenous fistula presenting with proximal vascular stenosis and prolonged bleeding time post-fistula needle removal due to heparin administration, the use of calcium-free dialysate facilitated heparin avoidance and mitigated bleeding time to a mere 10 min. Thus, this technique could offer respite for chronic hemodialysis patients grappling with vascular access complications, thereby alleviating patient burden.

Nevertheless, long-term studies are warranted to ascertain potential implications of prolonged calcium supplementation on calcium mass balance, parathyroid function, and the risk of soft tissue and blood vessel calcifications [21].

## 5. Conclusions

Our results confirm the efficacity and safety of calcium-free citrate containing dialysate, with calcium levels within the normal range throughout the dialysis session, requiring no adjustment in the calcium infusion rate. Thus, the adaptation of calcium perfusion to ionic dialysance if the vascular access is adequate does not seem necessary but will need to be confirmed by a prospective study. Likewise, the consequences of the long-term use of the technique on the phosphocalcic metabolism must be studied.

A particularity of our study is that we used the technique not only for patients with temporary heparin contraindication but also for a chronic dialysis patient. Thus, patients who experience vascular access complications such as prolonged bleeding time could benefit from this technique in a chronic setting. The stability of calcium levels throughout the session could imply that there is no need for systematic measurements at each session, but only once per week, if patients do not develop signs of hypo- or hypercalcemia.

## Figures and Tables

**Table 1:** Characteristics of the 52 patients and dialysis sessions.

Patients	*n* = 52
**Total Sessions**	**79**
**Sessions complicated by coagulation of dialysis circuit**	**7 (9%)**
Vascular access dysfunction	6 (7%)
Coagulation of dialyzer	1 (2%)
**Anticoagulation contraindications**	
Active bleeding	37 (47%)
Post-surgical intervention	39 (49%)
Organ biopsy	3 (4%)
**Vascular access**	
Arteriovenous fistula	49 (62%)
Catheter	30 (38%)
KT/V	1.42 ± 0.23
Dialysate flow (mL/min)	500
Blood flow (mL/min)	300 [200; 380]
Total ultrafiltration (L)	1.7 ± 0.9
Session Time (min)	240 [150; 300]

**Table 2:** Variations in ionized and total serum calcium at T0, T1, and end of the session.

	Prefilter at T0	Prefilter at T + 60 min	Prefilter at the End	*p*-Value
**Mean ionized calcium (mmol/L)**	1.08 ± 0.08	1.08 ± 0.08		0.96
**Mean ionized calcium (mmol/L)**	1.08 ± 0.08		1.19 ± 0.13	<0.05
**Mean total calcium** **mmol/L**	2.18 ± 0.16		2.46 ± 0.26	<0.05
**Mean** **total/ionized calcium ratio**	2.02 ± 0.12		2.06 ± 0.16	0.08

**Table 3:** Variations in ionized and total serum calcium in the case of our chronic hemodialysis patient.

	Prefilter at T0	Prefilter at T + 60 min	Prefilter at the End	*p*-Value
**Mean ionized calcium mmol/L**	1.02 ± 0.03	1.03 ± 0.05		<0.05
**Mean ionized calcium mmol/L**	1.02 ± 0.03		1.11 ± 0.09	<0.05
**Mean total calcium** **mmol/L**	2.14 ± 0.06		2.45 ± 0.16	<0.05
**Mean** **total /ionized calcium ratio**	2.09 ± 0.08		2.21 ± 0.19	<0.05

## Data Availability

All the data are available upon request from the corresponding author.

## Supplementary Materials

The following supporting information can be downloaded at https://www.mdpi.com/article/10.3390/jpm14060660/s1, Table S1: Hemodialysis Protocol with a Calcium-Free Dialysate, Grenoble University Hospital; Table S2: Global Thrombosis Index (GTI) Score.
