# Supplementary material for: Calcium-Free Dialysate Hemodialysis: A Simplified Approach
Source: J Pers Med. 2024 Jun 20;14(6):660. doi: 10.3390/jpm14060660 (PMC11204440; doi:10.3390/jpm14060660)
Supplement: Supplementary file 1 [file jpm-14-00660-s001.zip › jpm-3047283-supplementary.pdf]

Supplementary files :

Table S1: Hemodialysis Protocol with a Calcium-Free Dialysate, Grenoble University Hospital

I- Materials:
- Dialysis generators with measurement of ionic dialysance if possible
- Calcium chloride vial
- Citrate containing calcium-free dialysate: CITRASATE 484 Hemotech Ramonville
- If the patient has an arteriovenous fistula, 16 G and 15 G needles to allow a good blood flow

II- Prescription of the HD session with Calcium free citrate containing dialysate:

1- Session parameters:
- **Dialysate flow**: 500 ml/min
- **Blood flow**: Start the session with a minimum flow of 250 ml/min on AVF or catheter
- **Dialysate**: CITRASATE without calcium Hemotech (France)
  . Potassium: 2 mmol/l
  . Glucose: 1 g/l
  . Acetic acid: 0.3 mmol/L
  . Magnesium: 0.5 mmol/l
  Citric acid: 0.8 mmol/l
  Calcium: 0 mmol/l
- **Ultrafiltration**: according to patient's dry weight + 50 ml of UF/hour of session (200 ml for 4 hours)
- **High Permeability dialysis membrane**: No difference
- **If isolated ultrafiltration is prescribed**: it must be carried out within the first hour of the session, without the calcium infusion. Calcium infusion is started when switching to "hemodialysis" mode.

2- Reinfusion of calcium chloride:

a- Reinfusion solution:
- 20 vials of 10 ml of 10% calcium chloride , that is 200 ml
- Each 10 ml $CaCl_2$ vial contains 4.54 mmol of elemental calcium
- The concentration of the calcium chloride solution is 454 mmol/L

b- Reinfusion site:
- Directly at the level of the venous needle due to a "Y" connector between the venous needle and the venous line
- No reinjection into the venous bubble trap because blood stasis in the presence of air increases the risk of coagulation and clot formation in the bubble traps.

c- Reinfusion flow:

The calcium reinfusion rate is initially fixed for all patients at 42ml/h or 4.2g/h (for a dialysate calcium equivalent of 1.5 mmol/L).

The flow rate of the calcium infusion can then be adapted to the ionic dialysance according to the following chart:

Dialysance (ml/min)	Calcium chloride rate (ml/h)
50	10
60	12
70	14
80	16
90	18
100	20
110	22
120	24
130	26
140	28
150	30
160	32
170	34
180	36
190	38
200	40
210	42
220	44
230	46
240	48
250	50
260	52
270	54
280	56
290	58
300	60
310	62
320	64
330	66
340	68
350	70

Ensure that the calcium infusion never stops !!!

NB: Stop Calcium infusion only if there is a dialysate conductivity alarm with dialysate bypass otherwise risk of hypercalcemia

Calculation of the Calcium chloride reinfusion rate:
According to the work of Ridel et al. [5], the amount of calcium reinfusion according to ionic dialysance is derived from the following mass transfer equation:
We assume that JHDC = JRCA
- JHDC = net quantity of calcium provided by a conventional hemodialysis session at the end of dialysis
- JRCA = net quantity of calcium provided by the hemodialysis technique with a citrate containing calcium-free dialysate at the end of dialysis = Difference between the quantity of calcium infused into the patient and the loss of calcium with a calcium- free dialysate used throughout the session.
- Calcium losses linked to ultrafiltration are neglected

JHDC = DI x (Cd - Cp) = J Ca diffused dialysate-blood – J Ca diffused blood-dialysate
- DI is online ionic dialysance (mL/min)
- Cp is plasma ionized calcium (mmol/L)
- Cd is the calcium concentration of the dialysate (1.25 or 1.5 or 1.75 mmol/L)

JRCA = Qr x Cr - DI x Cp = J Ca infused - J blood-dialysate
- Qr = Injection rate of the compensation solution
- Cr = calcium concentration of the compensation solution

Thus, JRCA = Qr x Cr - DI x Cp = DI x (Cd - Cp)
We conclude that: Qr (ml/min) = DI x (Cd/Cr)
Qr (ml/h) = DI x 60 x (Cd/Cr)

However (Cd/Cr) x 60 is a constant R.
Thus, Qr = DI x R depends only on the ionic dialysance. Consequently, there is an interest of a flow chart to adapt the calcium perfusion rate according to each 10 ml/min variation in ionic dialysance.

3- Targets to be achieved and sampling site:

a- Targets to achieve:

- Patient's ionized serum calcium = Ca^{2+} > 1.1 and <1.35 mmol/l
- Ionized calcium level of the circuit = 0.15 to 0.4 mmol/l
- Patient's total calcium = 2.1 to 2.5 mmol/l

b- Sampling site:

- Patient's ionized calcium level: Arterial line before blood pump
- Ionized calcium level of the circuit: Venous line in post filter before the calcium chloride compensation solution

<u>III- Adverse Effects:</u>

1- Hypocalcemia:
Clinical signs :

. Perioral and extremities paresthesias
. Headache, dizziness, nausea, vomiting, abdominal pain
. Arterial hypotension, muscle twitching, muscle cramps
. Laryngospasm, bronchospasm, convulsions
. Neuromuscular signs:

. Trousseau sign: contraction of the hands triggered by relative ischemia of the upper limb by inflation of a blood pressure cuff to a few mmHg above the patient's systolic blood pressure for 3 min

. Chvostek sign: contraction of the perioral muscles triggered by percussion of the facial nerve at the level of zygomatic arch

If in doubt or in the event of clinical hypocalcemia: Call the doctor !

- Measure the ionized calcium of the patient and the machine in emergency
- Check that you have not forgotten to reinject calcium

If this is observed less than 30 minutes after connection, immediately adapt the calcium syringe to the prescribed flow rate

If this is observed more than 30 min after connection, prepare 1 vial of 10% calcium chloride to be infused in 5 min after medical approval at the venous line

- Consider changing the dialysate with a bath rich in calcium at 1.75 mmol/l and stopping the CITRASATE protocol for the rest of the session after medical approval.
- Then repeat a measurement of ionized calcium after medical approval
- Do an ECG = Prolongation of the QT space by ST prolongation, bradycardia, AV block, ventricular fibrillation

2- Hypercalcemia:

Clinical signs :

. Headache, mental confusion, drowsiness

. Osteotendinous hyporeflexia, distal paralysis, hypotonia and muscle weakness

. Agitation, delirium, hallucinations

. Nausea, vomiting, epigastric pain.

. Hypertension, tachycardia, sweats

If in doubt or in the event of clinical hypocalcemia: Call the doctor !

- Check the dialysate (put the calcium- free dialysate if not done)
- Measure the ionized calcium of the patient and the machine in emergency
- Stop the calcium infusion for 20 min
- Do an ECG = Shortening of the QT space by ST shortening, PR prolongation, moderate flattening of the T wave, tachycardia, ventricular fibrillation
- Then repeat the ionized calcium measurement after 20 min
- If serum calcium normalizes, resume calcium reinfusion at the speed initially prescribed
- If it remains high, recheck at 20 min without resuming reinfusion

<u>IV- Monitoring:</u>

1- Ionized Calcium Monitoring Rate:

- At connection: Patient's ionized + total calcium
- After 60 minutes: Patient’s ionized calcium + circuit ionized calcium
- At 240 min: Patient's ionized + total calcium

2- Clinical monitoring: every 60 minutes

. Ionic dialysance if possible (provided by the dialysis generator) in order to adapt the calcium perfusion

. AP, VP and TMP provided by the generator

. Blood pressure and heart rate

If the PTM is greater than 150 mmHg, this indicates the beginning of coagulation of the membrane requiring a change of circuit.

3- Monitoring of circuit coagulation:

After rinsing the circuit with saline solution, the degree of clotting and/or fibrin formation can be quantified according to the visual scale "Global Thrombosis Index".

Table S2: Global Thrombosis Index (GTI) Score

GTI score	Clear	Fibrin formation	Clotting
**Circuit lines**	0	0.5	1
**Dialyser**	0	0.5	1
**Venous bubble trap**	0	0.5	1
**Total GTI**			3
